# Estimation of Dietary Iron Bioavailability from Food Iron Intake and Iron Status

**DOI:** 10.1371/journal.pone.0111824

**Published:** 2014-10-30

**Authors:** Jack R. Dainty, Rachel Berry, Sean R. Lynch, Linda J. Harvey, Susan J. Fairweather-Tait

**Affiliations:** 1 Institute of Food Research, Norwich Research Park, Norwich, United Kingdom; 2 Department of Internal Medicine, Eastern Virginia Medical School, Norfolk, Virginia, United States of America; 3 University of East Anglia, Norwich Medical School, Norwich Research Park, Norwich, United Kingdom; University of Florida, United States of America

## Abstract

Currently there are no satisfactory methods for estimating dietary iron absorption (bioavailability) at a population level, but this is essential for deriving dietary reference values using the factorial approach. The aim of this work was to develop a novel approach for estimating dietary iron absorption using a population sample from a sub-section of the UK National Diet and Nutrition Survey (NDNS). Data were analyzed in 873 subjects from the 2000–2001 adult cohort of the NDNS, for whom both dietary intake data and hematological measures (hemoglobin and serum ferritin (SF) concentrations) were available. There were 495 men aged 19–64 y (mean age 42.7±12.1 y) and 378 pre-menopausal women (mean age 35.7±8.2 y). Individual dietary iron requirements were estimated using the Institute of Medicine calculations. A full probability approach was then applied to estimate the prevalence of dietary intakes that were insufficient to meet the needs of the men and women separately, based on their estimated daily iron intake and a series of absorption values ranging from 1–40%. The prevalence of SF concentrations below selected cut-off values (indicating that absorption was not high enough to maintain iron stores) was derived from individual SF concentrations. An estimate of dietary iron absorption required to maintain specified SF values was then calculated by matching the observed prevalence of insufficiency with the prevalence predicted for the series of absorption estimates. Mean daily dietary iron intakes were 13.5 mg for men and 9.8 mg for women. Mean calculated dietary absorption was 8% in men (50^th^ percentile for SF 85 µg/L) and 17% in women (50^th^ percentile for SF 38 µg/L). At a ferritin level of 45 µg/L estimated absorption was similar in men (14%) and women (13%). This new method can be used to calculate dietary iron absorption at a population level using data describing total iron intake and SF concentration.

## Introduction

Iron absorption in humans is dependent on physiological requirements, but may be restricted by the quantity and availability of iron in the diet [1], [2]. Dietary intake data reported in the present paper were collected from a 7d weighed intake, which is generally considered to be the minimum recording period necessary to achieve an accurate estimate of iron intake [3], [4]. The physiological regulation of absorption, determined by the size of the iron stores and the extent of erythropoietic activity, is responsible for maintaining iron balance. Levels of body iron in individuals with normal erythropoiesis are the main determinant of the efficiency of iron absorption, with serum ferritin (SF) being a well-established quantitative measure of iron stores, in healthy people [5], [6]. Methods for estimating bioavailability are more complicated than the assessment of iron intake. The diets of omnivores contain relatively small quantities of heme iron derived from meat and fish, which is always well absorbed, although iron status has a modest regulatory role [7], [8]. The remainder of the soluble iron forms a common non-heme iron pool and absorption is very variable, depending on meal composition, but its absorption is tightly regulated by iron stores [1].

Dietary iron fortification is generally considered the most cost effective method for reducing the prevalence of iron deficiency in populations that consume diets containing suboptimal quantities of bioavailable iron [9] and WHO/FAO recommends that the level of fortification is based on the estimated daily iron intake deficit adjusted for bioavailability [10]. There are no satisfactory methods for estimating dietary iron absorption at the population level, and therefore a qualitative assessment was used by WHO/FAO to assign one of three bioavailability levels (5%, 10% or 15%) [10]. In this paper we describe an alternative approach which could provide more accurate estimates of dietary iron absorption (bioavailability) that are relevant to specific target populations. The distribution of individual iron requirements is based on figures published by the Institute of Medicine (IOM) for menstruating women and men [11]. A full probability approach was used to predict the prevalence of an iron intake that would be sufficient to maintain iron balance based on estimated iron intake and a series of % absorption values from 1–40%. An estimate of average dietary absorption in the population sample was then calculated for selected SF concentrations by matching the observed prevalence of inadequacy (prevalence of SF below the designated level) with the prevalence predicted for the series of absorption estimates.

## Materials and Methods

Dietary iron intake and SF data from a previously published study were used. The methods for data collection are described briefly here, but have been published in greater detail elsewhere [12]. Data were collected as part of the National Diet and Nutrition Survey (NDNS) of adults aged 19–64 y living in private households in the UK between July 2000 and June 2001 [13], [14]. Approval for the survey was obtained from the South Thames Multi-Centre Research Ethics Committee (MREC) in 2000, with subsequent local approvals gained from 93 National Health Service Local Research Ethics Committees, which covered the 152 geographical areas selected for the fieldwork. Written informed consent was obtained from each participant for the clinical aspects of the study. The survey, conducted in a nationally representative sample of adults who were not pregnant or breastfeeding at the point of recruitment, involved an interview, a 7d dietary diary and blood and urine samples. Dietary intake was assessed using the 7d (consecutive) dietary record diary, with respondents recording all food and drink consumed in and out of the home. Following completion of the dietary survey, participants were interviewed to clarify and resolve difficulties, establish whether eating patterns were usual, and identify any illness during the recording period. Diaries were checked for omissions and level of detail (including brand details of pre-packed items) to ensure that foods could be accurately coded for nutrient analysis. After conversion of portion sizes to weights and subsequent coding of the diaries, the information was linked to the nutrient databank compiled by the Food Standards Agency. Quantities of nutrients ingested, including iron (total, heme and non-heme), were calculated from foods consumed. A total of 1347 (out of 2251) NDNS respondents provided a non-fasted blood sample, which was used for a range of hematological analyses.

α-1-antichymotrypsin (α1-ACT), an acute phase reactant, was measured because serum ferritin can be elevated in inflammatory conditions. The aim of this was to exclude any individuals with elevated serum ferritin due to inflammation/infection because the serum ferritin concentration would not be an accurate reflection of iron stores. C-reactive protein (CRP), a more commonly used indicator of inflammation, was not measured in the NDNS. Individuals consuming iron supplements were also excluded. Menopausal status was determined through information collected during the NDNS. Women who had entered menopause or were unsure of their status were excluded from the analytical sample because menstrual losses are highly variable at the onset of menopause. The final analytical sample of 873 subjects consisted of individuals with both dietary intake data and relevant hematological data (hemoglobin and SF). There were 378 premenopausal women aged 35.7±8.2 y and 495 men aged 19–64 y. Iron deficiency, anemia, and iron deficiency anemia were defined according to the WHO cut-offs: anemia, Hb<12.0 g/dL for women and <13.0 g/dL for men; iron deficiency, SF<15.0 ug/L for both men and women; iron deficiency anemia, Hb<12.0 g/dL and SF<15.0 ug/L for women, Hb<13.0 g/dL and SF<15.0 ug/L for men [15].

SF was measured on an Abbott IMx semi-automated analyzer using a standard Microparticle Enzyme Immunoassay (MEIA) kit. Sample concentrations were determined by comparison with a standard curve constructed from known concentrations. Quality control procedures comprised both internal and external procedures, with an internal pooled serum sample used as a drift control with each run. Hemoglobin concentrations were measured using a Bayer H3 Haematology Analyzer using a colorimeter at wavelength 546 nm. Quality control comprised both internal and external procedures. Daily commercial controls (Bayer Testpoint Haematology control) were used to monitor drift in all parameters. External quality assessment schemes included the National External Quality Assessment Scheme (NEQAS) for hematology and External Quality Assessment Scheme (EQAS) for hematology run by Addenbrookes Hospital, Cambridge, UK [14].

The Institute of Medicine (IOM) employed factorial modeling to calculate the distribution of estimated iron requirements needed to meet body functions with a minimal store for several age and sex groups including pre-menopausal women and men [11]. The values for pre-menopausal women (mixed population of oral contraceptive (OC) users and non-users) and men were used to derive the distributions of requirements for the NDNS study sample. Values reported as dietary intake requirements were converted to requirements for absorbed iron by multiplying by 0.18 (IOM values assume 18% absorption) [11]. The factorial model used by the IOM was designed to provide an estimate for individuals who were not anemic, but had very little storage iron. Our analysis was extended to include estimates of requirements needed to maintain selected levels of storage iron as defined by SF concentration. These estimates are valid because iron excretion is not increased when the iron stores accumulate, but remain in the physiological range [16], and iron balance is restored by a reduction in absorption. By using a spline function in the statistical package, R [17], it is possible to interpolate these values to derive the probabilities of inadequate iron absorption for 0.05 increments between 0 and 1. The latter values can then be used as a look-up table in a Microsoft Excel spreadsheet (**Table S1. Individual Data**) and compared to each individual’s absorbed iron estimate, based on their known iron intake and a theoretical range of iron absorption values (1–40%). The percentage absorption value that met the threshold for estimated requirement for the individual was designated as the dietary iron absorption for that individual. The average dietary absorption for the population was calculated as the mean of individual estimated absorption values. Subtracting the value found above from 100% gives the estimated percentage of the population who require more than this percentage of iron absorption (i.e. a higher bioavailability) to meet their requirements, or, in other words, the estimated prevalence of inadequate iron intakes. The population dietary iron absorption can be estimated for any SF concentration by assuming that the estimated prevalence of inadequate absorption calculated above is equivalent to the observed prevalence of iron insufficiency, as defined by the percentage of the population with a SF below the designated cut-off value.

## Results, Discussion, and Conclusions

**Figure 1:**
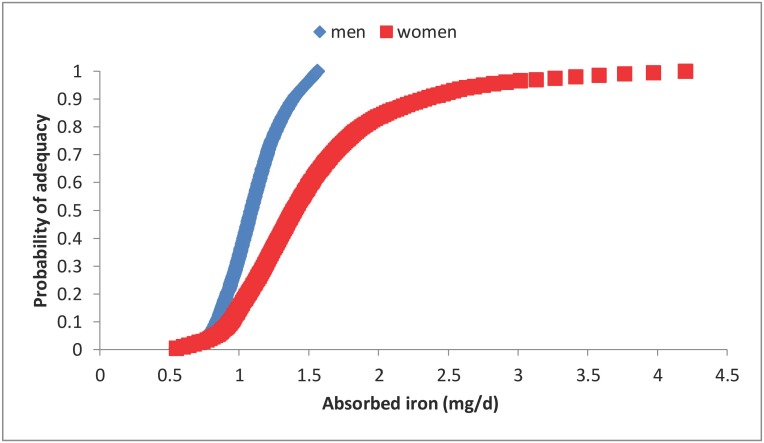
Distribution of estimated iron requirements for men (♦) and women (▪): y axis represents the probability of adequacy (0–1), x axis is absorbed iron (mg/d). This is based on tabulated data from the IOM [11]. The figure shows an interpolation of this data that was estimated using a spline function in R [17].

The distributions of estimated daily iron requirements for men and pre-menopausal women published by the IOM were used for the analyses described in this study (**Figure 1**) [11]. The IOM assumed that 17% of women were OC users. The percentage of menstruating women using OCs in the UK is estimated to be 25% [18], but this includes 16–18 year olds who are not part of our analytical sample. Although the NDNS survey included questions on contraceptive use, the answers were self-reported, and a large proportion of those practicing contraception did not answer the question on the method of contraception. A study across five European countries reported that the main method of contraception was OCs in 30% of the population with usage being even higher in the younger age groups [19]. It therefore appears that the IOMs assumption about OC use when calculating menstrual iron losses may have resulted in an overestimate of iron requirements since OCs reduce menstrual blood loss.

**Figure 2:**
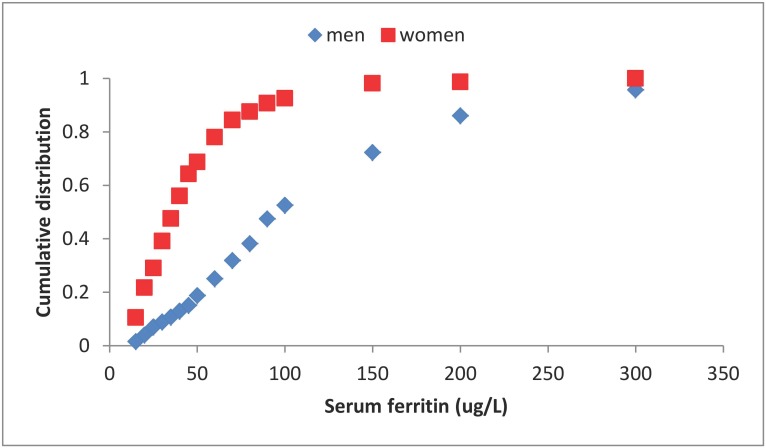
Cumulative distribution of serum ferritin concentrations for men (♦) and women (▪). The data from the NDNS survey [13], [14] are described in the Materials and Methods section (Men, n = 495; Women (pre-menopausal), n = 378).

**Figure 3:**
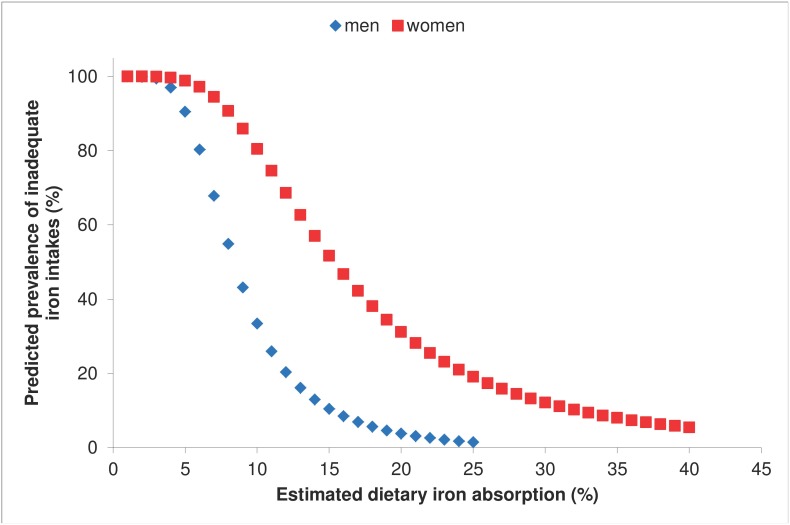
Results of probability modelling with NDNS data for men (♦) and women (▪): y axis represents the predicted prevalence of inadequate intakes (0–100%), x axis is estimated dietary iron absorption (%).

The NDNS sample was a relatively iron sufficient population (**Table 1**); the distributions of SF values for each of the two groups are shown in **Figure 2**. No individuals were identified with high levels of the inflammatory marker, α1-ACT (>0.65 g/L). Mean total iron intake was 13.5 mg, and 9.8 mg for and men and women respectively. The relationship between the arbitrary series of iron bioavailability values and the capacity of the diet to meet the iron requirements of men and women is shown in **Figure 3**. By comparing this figure with the cumulative distributions of SF values in the same population samples (Figure 2), it is possible to identify the average dietary absorption required to sustain a selected average iron status (as defined by the SF concentration) in the population. For example, estimated dietary absorption was 13% in women and 14% in men with SF values of 45 µg/L, and it was 31% for women with depleted iron stores (SF <15 µg/L) (**Table 2**). There were too few iron deficient men to allow a similar estimate for men to be calculated.

**Table 1:** Summary statistics for iron intake and status of the population sub-sample.

Group	n	Variable	Mean	SD	Lower95% CI	Upper95% CI
Pre-menopausalwomen	378	Age (y)	35.7	8.2	34.9	36.5
		Weight (kg)	68.1	14.4	66.6	69.5
		BMI (kg/m^2^)	26.0	5.5	25.4	26.5
		Iron intake (mg/d)	9.8	3.8	9.4	10.2
		Serum ferritin (µg/L)	45.5	38.4	41.7	49.4
		Hemoglobin (g/dL)	13.3	1.0	13.2	13.4
		Anemia (%)^1^	7.7			
		Iron deficient (%)^1^	12.4			
		Iron deficiency anemia(%)^1^	3.7			
Men	495	Age (y)	42.4	12.1	41.4	43.5
		Weight (kg)	83.7	14.1	82.5	85.0
		BMI (kg/m^2^)	27.1	4.3	26.7	27.5
		Iron intake (mg/d)	13.5	5.1	13.0	13.9
		Serum ferritin (µg/L)	121.6	112.1	111.7	131.5
		Hemoglobin (g/dL)	15.1	1.1	15.0	15.2
		Anemia (%)^1^	2.6			
		Iron deficient (%)^1^	2.0			
		Iron deficiency anemia (%)^1^	0.6			

1 Iron deficiency, anemia, and iron deficiency anemia defined according to the WHO cut-offs (15). Anemia: Hb<12.0 g/dL for women and <13.0 g/dL for men. Iron deficiency: SF<15.0 µg/L for both men and women. Iron deficiency anemia: Hb<12.0 g/dL and SF<15.0 µg/L for women, Hb<13.0 g/dL and SF<15.0 µg/L for men.

**Table 2:** Estimated dietary iron absorption for selected serum ferritin values in men and women.

1Serum ferritin cutoff(µg/L)	Probability model women(%)	Probability modelmen (%)	2Ratio method (%)
60	11	11	10
45	13	14	13
30	18	16	20
15	31		39

1 A serum ferritin cut off of 15 µg/L was used by the IOM to identify iron deficient individuals [11], 30 µg/L was used by Reddy et al. [25] for estimating non-heme iron bioavailability from meal composition, 60 µg/L is the value above which no homeostatic up-regulation of iron absorption occurs [40].

2 Estimated bioavailability adjusted for the effect of iron stores based on the ratio 45/SF cutoff.

The direct correlation between SF concentration and % non-heme iron absorption is well established [1], [20]–[22]. The results of iron absorption studies using isotopic labels are therefore usually corrected for the effect of iron status by adjusting absorption values. One method involves the inclusion of a “reference dose” in the study design, customarily 3 mg of highly bioavailable ferrous sulfate mixed with ascorbic acid [2], [23]. The observed absorption from the test meal is corrected to a mean reference value of 40%, which corresponds to absorption by individuals with borderline iron stores. This is made by multiplying test meal absorption values by 40/R where R is the reference dose absorption [2]. Another widely-used approach (ratio method) is to correct the measured absorption to a selected SF value by using the following equation:
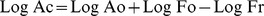
where Ac is the corrected dietary absorption, Ao is the observed absorption, Fo is the observed SF and Fr is the reference SF value selected. Values of 40 ug/L and 30 ug/L have been employed as the reference SF value [24], [25].

We applied the second method that adjusts absorption according to the SF concentration to our data. A SF of 45 ug/L was chosen as Fr and dietary iron absorption (bioavailability) was calculated for an arbitrary series of SF values that fall within the range of interest for population assessments (**Table 2**). There was reasonable agreement when the effect of iron status was adjusted using SF ratios and absorption estimates derived from the current probability model. Iron stores of men are significantly higher than those of menstruating women. The mean calculated dietary bioavailability (50^th^ percentile) in our sample was 8% for men (SF 85 ug/L) and 17% for women (SF 38 ug/L). However, as indicated above, when estimates were made for the same SF concentration, bioavailability was equivalent.

At a population level, dietary iron absorption is generally considered to be the most important determinant of iron status. There is, at present, no satisfactory method for estimating iron absorption from nutritional survey data. Algorithms based on the dietary factors that have been shown to affect non-heme bioavailability in isotopic absorption experiments (e.g. ascorbic acid, meat and fish, phytate, polyphenol, and calcium) have been developed to estimate bioavailability [26]. However, data from single meal studies exaggerate the effects of individual dietary factors on iron absorption [24]. The problem is compounded by the separate contribution of heme iron and the different effects of iron status on the absorption of non-heme and heme iron [7], [8]. Algorithms tend to underestimate bioavailability [27]. Two algorithms have recently been published that have been developed using data from studies in which non-heme iron absorption from whole diets was determined [28], [29]. However, these either require information about the intake of absorption promoters and inhibitors [28] or a value judgment must be made about the type of diet consumed in relation to its overall content of inhibitors or enhancers [29]. Program managers have therefore applied algorithms sparingly, and the approximations based on qualitative data (5%, 10%, 15%) quoted in the WHO guidelines [10] are more often used. An alternative approach is now suggested that just requires data on total iron intake and measurements of iron status, and avoids the need to obtain information on dietary inhibitors and enhancers, which are notoriously difficult to collect. Its validity depends on three critical elements, and the samples selected for the development of the new approach described in this study meet all these criteria:

- The accuracy of the estimation of the distribution of individual iron requirements. In adults, iron requirements are calculated from measured losses. Estimates for men are based on a factorial approach employing experimental measurements which are relatively precise and unlikely to vary in different population samples [16], [30]. It is more difficult to obtain accurate estimates of menstrual losses, which are an important component of the requirements of pre-menopausal women. However, carefully controlled measurements in several population samples have yielded surprisingly consistent distributions of menstrual blood losses [11], [31], [32]. OCs reduce menstrual blood loss while intrauterine contraceptive devices tend to increase menstrual bleeding [33]. The increasing use of OCs may have led to a modest overestimate of the requirement for menstruating women and therefore dietary iron absorption by the probability approach in the current study. Nevertheless there was good agreement between estimates for men and women at the same SF concentration.
- The accuracy of dietary intake data. This new method requires an accurate estimate of habitual total iron intake from all sources, including non-heme and heme iron, but information on other dietary constituents (i.e. enhancers and inhibitors of iron absorption) that may be more difficult to estimate is not required.
- A stable iron intake. Hallberg et al. [34] calculated the rate of change in iron stores following changes in dietary intake. It takes about 2 years to reach a new balanced state, but 80% of the adjustment in absorption occurs within the first year. The method for calculating dietary bioavailability that we describe is therefore not applicable to children, women during pregnancy and lactation, or immediately after the onset of menopause because of the variability in iron requirements.

There is no formal agreement about the iron status for which iron absorption values should be reported as the dietary bioavailability. However, most investigators and the IOM have conceptualized bioavailability as the absorption value that would be attained by an individual who is not anemic, but has only a minimal quantity of storage iron. This is based on the assumption that physiological function remains normal after stores are exhausted if there is still sufficient iron to maintain the functional compartments. Thus, absorption is maximally up-regulated without any impairment of physiological processes. A serum ferritin value of 15 ug/L is the cut-off value selected by the WHO [15]. The IOM also defined bioavailability to be the estimated absorption in an individual with a serum ferritin concentration of 15 µg/L, as this has been reported to be the most reliable cut-off value for absent stainable iron in the bone marrow [35]. However, a higher iron status may have pragmatic advantages for setting optimal iron intake levels in populations. Although the iron store appears to have no functional importance, other than as a source of readily available iron if there is a sudden increase in requirements (e.g. as the result of blood loss), it may be desirable for individuals to have this safety net at all times. There is also some evidence to suggest that adequate iron status in early pregnancy is important for birth outcome [36], [37]. The proposed new approach for estimating dietary iron absorption (bioavailability) allows estimates to be made for any selected mean population serum ferritin level as shown in Figure 3.

The concordance between dietary absorption values derived from the two sub-samples, men and pre-menopausal women, who have very different iron requirements provides support for the validity of the estimates (Figure 3, Table 2). Furthermore, iron stores are known to be the primary physiological regulator of iron absorption in healthy adults. Adjustments for the effect of iron status using serum ferritin ratios (Table 2) yields results that are similar to those derived from the probability model although the ratio method predicts higher bioavailability in individuals with absent iron stores. The results derived from the probability model have a high degree of uncertainty in the lower range in our population samples because of the low prevalence of iron deficiency. It is therefore not possible to comment on possible explanations for the difference, and the analysis of data from regions where nutritional iron deficiency is common would be informative. Finally, it is important to note that the probability approach predicts the potential for a higher bioavailability (31%) than that employed in calculating the IOM Dietary Reference Intakes (18%) in iron deficient individuals (serum ferritin <15 ug/L) [11].

The method that we have described has several strengths. It is based on experimental data drawn from the target population. Dietary assessment is relatively simple; it is only necessary to determine the total iron intake, not the intake of dietary enhancers and inhibitors of iron absorption, and there is no need to estimate heme iron intake. The dietary iron absorption needed to achieve a desired iron store in a target population can be calculated, or alternatively the necessary level of fortification can be calculated based on these estimates. However, there are some weaknesses of the method. Iron requirements and iron intake must be in a steady state (for at least one year), therefore the method cannot be used for children, pregnant women, and immediately after the menopause because of changing requirements. The increasing use of oral contraceptives may reduce menstrual iron loss and therefore currently available estimates of iron requirements [10]. Finally, care must be taken to ensure that estimates of iron status based on serum ferritin concentration are not confounded by inflammation/infection or obesity [38], [39].

## Supporting Information

Table S1 **Individual Data.** Excel file containing individual data for adults in the UK National Diet and Nutrition Survey (men aged 19–64 y and women aged 20–49 y): serum ferritin (µg/L), daily iron intake (mg), calculated quantity of iron absorbed (mg/d) at efficiencies of absorption ranging from 1–25%, estimated physiological requirements of iron to replace obligatory losses (mg/d), and predicted prevalence of inadequate intake at each % absorption efficiency.
(XLSX)
