# Correction: Estimation of Dietary Iron Bioavailability from Food Iron Intake and Iron Status

**DOI:** 10.1371/journal.pone.0316371

**Published:** 2024-12-20

**Authors:** Jack R. Dainty, Rachel Berry, Sean R. Lynch, Linda J. Harvey, Susan J. Fairweather-Tait

S1 Table is uploaded incorrectly. Please view the correct S1 Table below.

**Individual Data.** Excel file containing individual data for adults in the UK National Diet and Nutrition Survey (men aged 19–64 y and women aged 20–49 y): serum ferritin (µg/L), daily iron intake (mg), calculated quantity of iron absorbed (mg/d) at efficiencies of absorption ranging from 1–25%, estimated physiological requirements of iron to replace obligatory losses (mg/d), and predicted prevalence of inadequate intake at each % absorption efficiency.

## Supporting information

S1 Table (XLSX)
